# An Efficient and Recyclable Ionic Liquid-Supported Proline Catalyzed Knoevenagel Condensation

**DOI:** 10.5402/2011/676789

**Published:** 2011-04-10

**Authors:** Chen Zhuo, Dong Xian, Wu Jian-wei, Xie Hui

**Affiliations:** ^1^School of Chemistry and Material Science, Guizhou Normal University, Guiyang 550001, China; ^2^College of Pharmaceutical Sciences, Zhejiang University, Zijin Compus, Hangzhou 310058, China

## Abstract

The Knoevenagel condensation reaction of aldehydes with malononitrile was described in this study, which was catalyzed by an efficient and recyclable ionic liquid-supported proline. The method represented an attractive alternative to the classical synthesis strategies and exhibited the advantage of performing homogeneous chemistry on a large scale additionally avoided large excesses of reagents. The products were obtained in good yields and reasonable purities without the need for further chromatographic purification. Moreover, the catalyst could be reused for at least four times.

## 1. Introduction

The Knoevenagel condensation reaction is one of the most widely used methods for the preparation of substituted alkenes. However, many of these reactions exhibit one or more drawbacks, such as use of hazardous and carcinogenic solvents as well as poor recovery of the catalysts, which limit the wide application of these reactions in industrial processes [1]. Especially, ethanol and toluene, which are the most commonly used solvents in these condensation processes, have been classified as hazardous air pollutants in the TRI list [2, 3]. In order to resolve the disadvantages mentioned above, some novel reaction conditions have been developed, such as the use of solid catalyst based on inorganic supports [4–6] and solvent-free microwave-assisted conditions [7, 8]. Although the solid supports and microwave routes have shortened reaction time, the yields of desired alkenes are low with high variability (3–86%). Proline has been recently used as a catalyst for many kinds of reactions including Mannich reaction [9] and the direct asymmetric aldol reaction [10]. No metal derivatives are required for these reactions and the catalyst proline is cheap and readily accessible. However, the large-scale application of these procedures was limited due to the toxic and/or hazardous properties of the organic solvents used, including dimethyl sulfoxide, dimethylformamide, chloroform, or tetrahydrofuran [3].

Recently, ionic liquids have attracted considerable interest as environmentally benign reaction media due to their specific properties such as high thermal and chemical stability, no measurable vapor pressure, nonflammability, friction reduction, antiwearing performance, and high loading capacity [11, 12]. An attractive feature of ionic liquids is that their solubility can be tuned readily. Therefore, phase separation of ionic liquids from organic or aqueous media is allowed depending on the choice of cations and anions. In addition, in many cases, ionic liquids can be easily recycled [13–15]. Ionic liquid technology has been successfully applied in several classical organic processes and other procedures. The implementation of task specific ionic liquids further enhances the versatility of classical ionic liquids where both reagent and medium are coupled [16]. In our previous study, ionic liquid-supported proline was used to catalyze direct asymmetric aldol reaction [17]. Further, we investigated the catalytic activity of ionic liquid-supported proline for Knoevenagel condensation reactions and the regenerability.

## 2. Results and Discussion

**Scheme 1:**
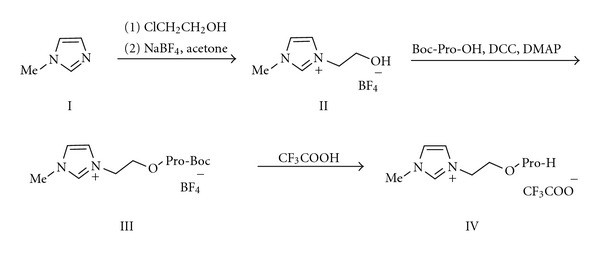
Synthesis of ionic liquid-supported proline.

**Scheme 2:**
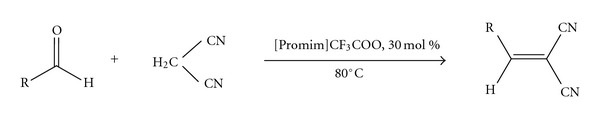
Knoevenagel condensation of malononitrile with varying aldehydes and carbonyl derivatives.

Ionic liquid-supported proline IV ([Promim]CF_3_COO) was synthesized following a similar route reported previously (Scheme 1) [17]. The [Promim]CF_3_COO (IV) was used to catalyze the Knoevenagel condensation of malononitrile with varying aldehydes and carbonyl derivatives (Scheme 2). The reactions were performed with 30 mol % of the catalyst for 24 h at 80°C, resulting in expected Knoevenagel condensation products in good yield (Table 1). We speculate that the enhanced rates result from IL-based stability of the imine electrophiles, and electron-donating groups of aromatic aldehyde can promote the stability of imine electrophile. An alternative lower energy reaction pathway is utilized when the ionizing solvent is present to assist in the formation and stabilization of separated charges.

Some interesting trends were observed regarding the resulting Knoevenagel condensation products. As shown in Table 1, higher condensation product yield was achieved for more reactive systems containing benzaldehyde or electron-deficient aryl aldehydes than the electron-rich anisaldehyde. Overall, aryl aldehydes (Entries 1–12) and the assayed conjugated aldehyde (Entry 14) gave better results than the assayed aliphatic aldehyde (Entry 15) under the same reaction conditions. In most cases, no elimination products were detected, or they were formed in insignificant amounts. Ionic liquid-supported proline IV is soluble in water and slightly soluble in alcohols and thus avoiding catalysts signals in the NMR spectra. Removing ionic liquid-supported proline IV could be accomplished by washing the IL-based with 5% aqueous ethanol. We isolated the expected Knoevenagel condensation products in good yields, compared previous reports have documented the application and reuse of IL technology in same organic processes [18, 19].

Next we examined the effect of the catalyst amount on Knoevenagel condensation of malononitrile with p-chlorobenzaldehyde. Some blank experiments were also carried out to demonstrate the catalysis of ionic liquid-supported proline. Reactions containing varying amounts of [Promim]CF_3_COO (IV) but without catalyst resulted in low yields. Comparable results were observed in the presence of 10 and 30 mol % of the catalyst (Table 2). 

We also examined the solvent effect in the ionic liquid-supported proline (IV) catalysis. The reaction of p-chlorobenzaldehyde with malononitrile in acetonitrile was selected as a model study. The effects of various solvents on the yield and purity of Knoevenagel condensation products were shown in Table 3. The successful results of experiments showed that the ionic liquid-supported proline (IV)-catalyzed Knoevenagel condensation in CH_3_CN is substantially better than the corresponding reaction in conventional organic solvents. The use of CH_3_CN resulted in the highest yield (96%), followed by CH_3_OH (90%), H_2_O (86%), CHCl_3_ (72%), and CH_2_ClCH_2_Cl (57%) under the same experimental conditions. 

Successive reuse of the recovered ionic liquid-supported proline (IV) in the same reaction yielded amounts of product as high as in the first cycle. The system of ionic liquid-supported proline (IV) was thoroughly extracted with ether to remove all organic impurities. Our aim was to recycle the IL medium, and using indicators such as mass balance and percent conversion in conjunction with NMR spectroscopy, examine the IL medium after each run in an effort to learn more about the application of this technology in modern synthetic transformations. As shown in Table 4, no considerable decrease in reactivity and yield was observed after four cycles when the same reaction time was strictly maintained. We do believe that the ionic liquid-supported proline serves as promoter for these reactions throughout the recycling study.

## 3. Experimental

The ^1^H NMR spectra were recorded on Brucker Avance DMX500MHz instrument with CDCl_3_ or dimethylsulfoxide (d^6^-DMSO) as solvent and tetramethylsilane (TMS) as the internal standard, and the chemical shifts are expressed in parts per million (ppm). The purity was determined by HPLC analyses with an Agilent 1100 equipment. The melting points of the synthetic products were determined on an X-4 meltingpoint measurement device.

### 3.1. Synthesis of Ionic Liquid-Supported Proline (IV)

Ionic liquid-supported proline, 1-Methyl-3-{2-[(2S)-pyrrolidine-2-carbonyloxy]-ethyl}-1H-imidazol-3-ium trifluoroacetate (IV), was prepared and indicated according to the literature [17].

### 3.2. General Procedure for Knoevenagel Condensation of Malononitrile with Aldehydes

To a mixture of the aldehydes (1 mmol) and malononitrile (1.1 mmol) in anhydrous acetonitrile (2 mL) was added 30 mol % ionic liquid-supported proline (IV). The mixture was stirred at 80°C for 24 h. The reactions were monitored by thin layer chromatography (TLC). Upon completion of the reaction, the solvent was removed under vacuum and the residue was extracted with ether (3 × 20 mL). The organic extract was then combined under reduced pressure, washing with 5% aqueous ethanol and filtering to give the desired products in high purity. After isolation of the product, the remainder of the ionic liquid-supported proline (IV) was dried under vacuum. The next run was performed under identical reaction conditions.

**(1) 2-Benzylidenemalononitrile**

Melting point: 80~82°C. ^1^H NMR (500 MHz, CDCl_3_) *δ* 7.53–7.56 (2H, m), 7.62–7.65 (1H, m), 7.78 (1H, s), 7.90–7.92 (2H, m).

**(2) 2-(4-Bromobenzylidene)malononitrile**

Melting point: 153~156°C. ^1^H NMR (500 MHz, CDCl_3_) *δ* 7.68–7.70 (2H, d, *J* = 9.0 Hz), 7.71 (1H, s), 7.76–7.78 (2H, d, *J* = 9.0 Hz).

**(3) 2-(4-Hydroxybenzylidene)malononitrile**

Melting point: 189~190°C. ^1^H NMR (500 MHz, CDCl_3_) *δ* 6.95–6.97 (2H, d, *J* = 9.0 Hz), 7.64 (1H, s), 7.87–7.89 (2H, d, *J* = 9.0 Hz).

**(4) 2-(4-Methoxybenzylidene)malononitrile**

Melting point: 114~115°C. ^1^H NMR (500 MHz, CDCl_3_) *δ* 3.92 (3H, s), 7.01-7.02 (2H, d, *J* = 9.0 Hz), 7.65 (1H, s), 7.90–7.92 (2H, d, *J* = 9.0 Hz).

**(5) 2-(4-(Dimethylamino)benzylidene)malononitrile**

Melting point: 180~182°C. ^1^H NMR (500 MHz, CDCl_3_) *δ* 3.14 (6H, s), 6.68–6.70 (2H, d, *J* = 9.0 Hz), 7.46 (1H, s), 7.81-7.82 (2H, d, *J* = 9.0 Hz).

**(6) 2-(4-(Trifluoromethyl)benzylidene)malononitrile**

Melting point: 96~98°C. ^1^H NMR (500 MHz, CDCl_3_) *δ* 7.80–7.82 (2H, d, *J* = 8.5 Hz), 7.84 (1H, s), 8.00–8.02 (2H, d, *J* = 8.5 Hz).

**(7) 2-(4-Chlorobenzylidene)malononitrile**

Melting point: 163~164°C. ^1^H NMR (500 MHz, CDCl_3_) *δ* 7.51–7.53 (2H, d, *J* = 8.5 Hz), 7.73 (1H, s), 7.85-7.86 (2H, d, *J* = 8.5 Hz).

**(8) 2-(3-Fluorobenzylidene)malononitrile**

Melting point: 88~89°C. ^1^H NMR (500 MHz, CDCl_3_) *δ* 7.45-7.46 (1H, m), 7.54-7.55 (2H, d), 8.17–8.19 (2H, d, *J* = 8.0 Hz), 8.27 (1H, s).

**(9) 2-(3-Chlorobenzylidene)malononitrile**

Melting point: 95~96°C. ^1^H NMR (500 MHz, CDCl_3_) *δ* 7.32–7.36 (1H, m), 7.52–7.56 (1H, m), 7.62–7.65 (1H, dd, *J* = 2.0 Hz), 7.67-7.68 (1H, d, *J* = 8.0 Hz), 7.75 (1H, s).

**(10) 2-(3-Nitrobenzylidene)malononitrile**

Melting point: 98~101°C. ^1^H NMR (500 MHz, CDCl_3_) *δ* 7.78–7.82 (1H, t), 7.90 (1H, s), 8.32–8.34 (1H, d, *J* = 9.5 Hz), 8.47–8.49 (1H, d, *J* = 9.5 Hz), 8.67 (1H, s).

**(11) 2-(2,4-Dihydroxybenzylidene)malononitrile**

Melting point: 243~246°C. ^1^H NMR (500 MHz, d_6_-DMSO) *δ* 6.79 (1H, s), 6.90-6.91 (1H, d, *J* = 2.0 Hz), 7.64–7.66 (1H, d, *J* = 9.0 Hz), 8.79 (1H, s).

**(12) 2-(3,4-Dimethoxybenzylidene)malononitrile**

Melting point: 149~152°C. ^1^H NMR (500 MHz, CDCl_3_) *δ* 3.95–3.99 (6H, d), 6.95–6.97 (1H, d, *J* = 8.5 Hz), 7.37–7.39 (1H, m), 7.64 (1H, s), 7.68-7.69 (1H, d, *J* = 2.0 Hz).

**(13) 2-(3-Phenylallylidene)malononitrile**

Melting point: 111~113°C. ^1^H NMR (500 MHz, CDCl_3_) *δ* 7.25-7.26 (2H, d), 7.45-7.46 (3H, m), 7.59–7.61 (2H, m), 7.79 (1H, s).

**(14) 2-(Furan-2-ylmethylene)malononitrile**

Melting point: 63~66°C. ^1^H NMR (500 MHz, CDCl_3_) *δ* 6.72 (1H, s), 7.37 (1H, s), 7.51 (1H, s), 7.81 (1H, s).

**(15) 2-(3-Phenylpropylidene)malononitrile**

Melting point: 127~129°C. ^1^H NMR (500 MHz, CDCl_3_) *δ* 2.88–2.90 (2H, m), 2.91–2.93 (2H, m), 7.16–7.18 (1H, m), 7.25–7.30 (3H, m), 7.32–7.36 (2H, m).

## 4. Conclusion

In summary, the ionic liquid-supported proline (IV) catalyzed Knoevenagel condensation reaction was successfully performed in this study with increased product yield. Our results demonstrate that ionic liquid-supported proline as catalyst provides a convenient approach to phase separation and isolation of the products, which may be used in other fields of organic synthesis. The structure and purity of the products were verified easily by routine spectroscopic analysis, and no chromatographic procedures were needed during the synthesis. Studies which include an examination of the role of the catalyst using optimized reaction conditions are currently being investigated and will be reported in due course.

## Figures and Tables

**Table 1:** Knoevenagel condensation of malononitrile with aldehydes in the presence of [Promim]CF_3_COO (IV).

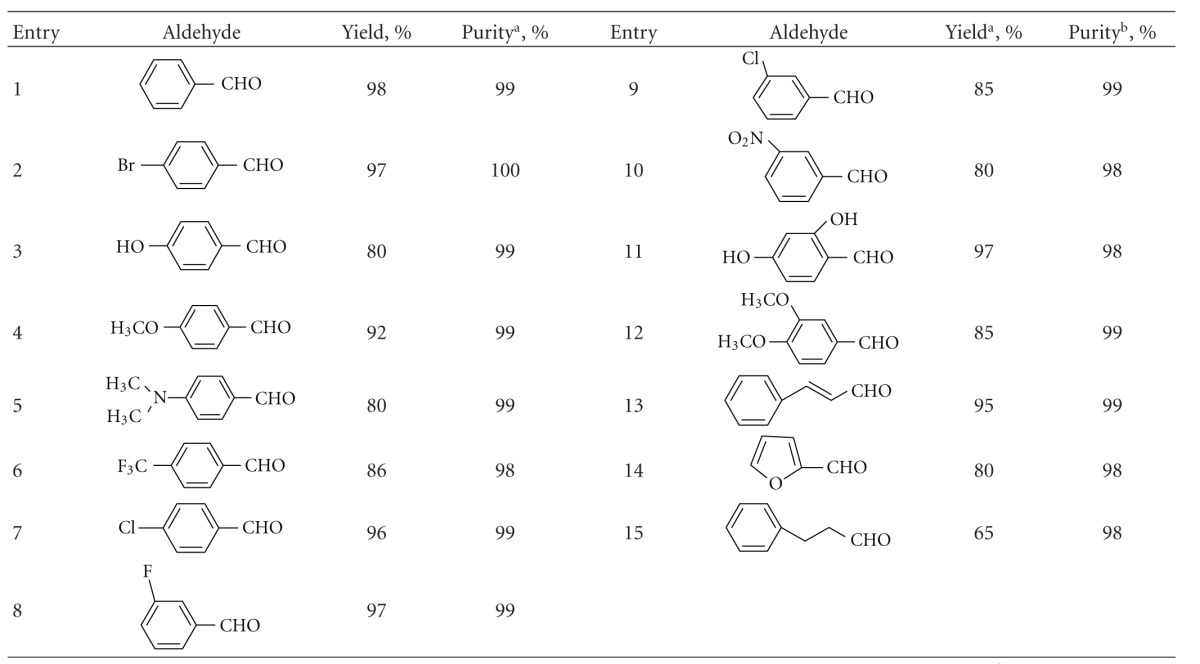

Reaction conditions: aldehydes: 1 mmol; malononitrile: 1.1 mmol; [Promim]CF_3_COO (IV): 30 mol %; 80°C, 24 h. ^a^: Isolated yield. ^b^: purity of the product was determined by HPLC.

**Table 2:** Effect of [Promim]CF_3_COO (IV) amount on Knoevenagel condensation.

Catalyst, mol %	Yield, %	Purity, %
0	40	99
10	81	99
20	90	99
30	96	99
40	92	99

Reaction conditions are the same with Table 1 based on p-chlorobenzaldehyde with malononitrile.

**Table 3:** Effect of the solvent on Knoevenagel condensation.

Entry	Solvent	Yield, %	Purity, %
1	CH_3_CN	96	99
2	CHCl_3_	72	99
3	CH_3_OH	90	99
4	H_2_O	86	99
5	CH_2_ClCH_2_Cl	57	99

Reaction conditions are the same with Table 1 based on p-chlorobenzaldehyde with malononitrile.

**Table 4:** Recycling of [Promim]CF_3_COO IV in the Knoevenagel condensation.

Cycle no.	Yield, %	Purity, %
1	96	99
2	94	99
3	96	99
4	86	99

Reaction conditions are the same with Table 1 based on p-chlorobenzaldehyde with malononitrile.
